# The psychoactive cannabinoid THC inhibits peripheral nociceptors by targeting Na_V_1.7 and Na_V_1.8 nociceptive sodium channels

**DOI:** 10.1038/s41386-026-02355-9

**Published:** 2026-01-21

**Authors:** Yossef Maatuf, Ariel Iskimov, Alexander M. Binshtok, Avi Priel

**Affiliations:** 1 The Institute for Drug Research, School of Pharmacy, Faculty of Medicine, The Hebrew University of Jerusalem, Jerusalem, Israel; 2 Department of Medical Neurobiology, Institute for Medical Research Israel-Canada, Faculty of Medicine, The Hebrew University of Jerusalem, Jerusalem, Israel; 3 The Edmond and Lily Safra Center for Brain Sciences, The Hebrew University of Jerusalem, Jerusalem, Israel

**Keywords:** Ion channels in the nervous system, Pharmacology

## Abstract

Δ⁹-Tetrahydrocannabinol (THC), the primary psychoactive compound in cannabis, is widely recognized for its central effects mediated by cannabinoid receptors. Here, we uncover a distinct peripheral mechanism by which THC inhibits the excitability of nociceptive neurons. We show that THC directly targets the nociceptive voltage-gated sodium channels Na_V_1.7 and Na_V_1.8 through the conserved local anesthetic binding site. This interaction reduces sodium currents and suppresses action potential generation in peripheral sensory neurons. Our findings demonstrate that, beyond its central psychoactivity, THC exerts direct peripheral nociceptor inhibition via modulation of Na_V_1.7 and Na_V_1.8, offering new insight into cannabinoid-based analgesia independent of cannabinoid receptor signaling.

## Introduction

Cannabis has been used for centuries for its analgesic properties, and its clinical relevance in pain management continues to grow [1, 2]. The primary psychoactive constituent of Cannabis sativa, Δ⁹-tetrahydrocannabinol (THC), produces central effects attributed mainly to its interaction with cannabinoid receptors, especially CB1, in the brain and spinal cord [3]. These receptor-mediated actions underlie much of the known psychoactive and analgesic activity of THC [4, 5]. However, recent studies have shown that non-psychoactive cannabinoids such as cannabidiol (CBD) and cannabigerol (CBG) modulate peripheral nociceptor activity by directly inhibiting voltage-gated sodium channels (Na_V_s), particularly Na_V_1.7 and Na_V_1.8 [6–11]. These channels, expressed in peripheral sensory neurons, play a central role in determining nociceptor excitability and are key targets for analgesic interventions, including local anesthetics that act via a conserved binding site [12–15]. While peripheral, receptor-independent mechanisms have been characterized for non-psychoactive cannabinoids, no direct peripheral mechanism of THC targeting either nociceptors or nociceptive Na_V_s has been previously established [16].

In this study, we identify a distinct peripheral mode of action for THC that is independent of cannabinoid receptor signaling. We show that THC directly interacts with hNa_V_1.7 and hNa_V_1.8 via the conserved local anesthetic binding site, leading to inhibition of sodium currents and suppression of action potential firing in nociceptive neurons. These findings reveal a previously unrecognized mechanism for THC-mediated peripheral analgesia and establish a non-canonical molecular pathway through which the psychoactive cannabinoid can inhibit nociceptor excitability and thereby pain.

## Materials and methods

### Animals

All animal procedures were carried out under protocols approved by the Hebrew University Ethics Committee (MD-20-16310-2 and HU-24-17640-1). Male and female Sprague–Dawley rat pups (Envigo, Jerusalem, Israel) at postnatal days 3–5 were used for neuronal isolations.

### Neuronal primary cell cultures

Primary trigeminal (TG) neurons were acutely dissociated from 3–5-day-old Sprague–Dawley rats, as previously described [15]. Briefly, ganglia were collected from 2–4 pups and placed in ice-cold DPBS. The tissue was enzymatically digested at 37 °C using 0.025% collagenase P (Sigma-Aldrich, USA) for 10 min, followed by 0.25% trypsin (Gibco, USA) for 5 min. Neurons were mechanically dissociated by gentle trituration using fire-polished Pasteur pipettes of decreasing tip diameters, and undissociated tissue was removed. After centrifugation, the cell pellet was resuspended in Leibovitz’s L-15 medium (Gibco, USA) supplemented with 10% fetal calf serum, 5 mM HEPES, 1% penicillin–streptomycin (Gibco, USA), and 100 ng/ml nerve growth factor (NGF; Alomone Labs, Israel). Cells were plated on glass coverslips coated with poly-D-lysine (PDL; Sigma-Aldrich, USA) and laminin (R&D Systems, USA) and incubated at 33 °C at room temperature. After 30 min, a complete L-15 medium was added, and cells were further incubated for 2–4 h at 33 °C in room air. Throughout this period, the neurons maintained healthy morphology and exhibited negative resting membrane potentials, as well as overshooting action potentials. No significant differences were observed in action potential or sodium current properties between cells used on the day of preparation and those stored at 4 °C for up to 48 h.

### Molecular biology

The cDNAs encoding the human voltage-gated sodium channel α-subunits—SCN1A (hNa_V_1.1; NM_001165963), SCN2A (hNa_V_1.2; NM_021007), SCN3A (hNa_V_1.3; NM_001081677), SCN4A (hNa_V_1.4; NM_000334), SCN5A (hNa_V_1.5; NM_198056), SCN8A (hNa_V_1.6; NM_014191), SCN9A (hNa_V_1.7; NM_002977), and SCN10A (hNa_V_1.8; NM_006514 each cloned into the pCMV6 expression vector, were obtained from Origene Technologies (USA). The sodium channel auxiliary subunit SCN1B (β1) cDNA was generously provided by Prof. Dr. Angelika Lampert (RWTH Aachen University, Aachen, Germany). The SCN3B (β3) auxiliary subunit cDNA was cloned from rat trigeminal ganglion (TG) neurons [15]. Site-directed mutagenesis of the hNa_V_1.8 cDNA was performed using the QuikChange II XL Site-Directed Mutagenesis Kit (Agilent Technologies, USA).

### Heterologous cell culture and channel expression

HEK293T and ND23/7 cells were cultured in DMEM (Gibco, USA) supplemented with 10% fetal bovine serum (Gibco, USA), 1% penicillin–streptomycin (Gibco, USA), and 25 mM HEPES (Gibco, USA) at 37 °C in a 5% CO₂ atmosphere. Transient expression of hNa_V_1.1–hNa_V_1.8 and mutant channel constructs was performed using Lipofectamine 3000 (Invitrogen, USA) according to the manufacturer’s instructions. hNa_V_1.1–hNa_V_1.7 channels were co-transfected with the β1 auxiliary subunit, while hNa_V_1.8 was co-transfected with the β3 subunit. At 24–48 h post-transfection, cells were transferred onto poly-D-lysine (PDL; Sigma-Aldrich, USA)-coated glass coverslips and used for whole-cell voltage-clamp recordings.

### Chemicals

Δ⁹-Tetrahydrocannabinol (THC) and Cannabidiol (CBD) were obtained from THC Pharm GmbH (Frankfurt, Germany) and prepared as stock solutions in ethanol (Carlo Erba, Italy) and DMSO (Sigma-Aldrich, USA), respectively. Cannabichromene (CBC) was purchased from Alomone Labs (Jerusalem, Israel), and Cannabigerol (CBG) was purchased from Symrise AG (Germany). Tetrodotoxin (TTX; Alomone Labs, Israel) was dissolved in molecular biology-grade water (Biological Industries, Israel). All stock solutions were stored at –20 °C until use and diluted in extracellular solution to achieve final working concentrations. To enhance drug solubility, Pluronic F-127 (20%) (Sigma-Aldrich, USA) was added to the final working solutions. The final concentrations of ethanol (≤1%), DMSO (≤1%), and Pluronic F-127 (0.01%) were confirmed not to affect action potentials or current properties. To isolate TTX-resistant (TTX-R) sodium currents, 100 nM TTX was included in the external and working solutions when recording from hNaV1.8 and mutant channels expressed in ND7/23 cells, as well as from TTX-R currents in trigeminal ganglion (TG) neurons.

### Electrophysiology

Nociceptive neurons were identified based on a soma diameter of ≤25 µm and further verified by their responsiveness to capsaicin and the functional expression of Na_V_1.7 and Na_V_1.8 nociceptive sodium channels. Neurons with soma diameters >35 µm were classified as non-nociceptive. Whole-cell patch-clamp recordings were performed as previously described [17–19]. Patch pipettes were pulled from borosilicate glass capillaries using a P-1000 micropipette puller (Sutter Instrument, USA) and then fire-polished with an MF-900 microforge (Narishige, Japan) to achieve a resistance of 2–5 MΩ. Membrane currents and potentials were recorded using an Axopatch 200B amplifier (Molecular Devices, USA), digitized with a Digidata 1440 A interface, and acquired using pCLAMP 10.6 software (Molecular Devices, USA). Signals were sampled at 10 kHz and then low-pass filtered at 1 kHz. Recordings began at least 10 min after achieving whole-cell configuration to ensure current stability. The leak current was continuously monitored throughout the experiments. TG neurons with a leak above ~100 pA were discarded from the analysis. During recordings, extracellular solutions were continuously perfused using the ValveBank II perfusion system (AutoMate Scientific, USA). All experiments were conducted at room temperature.

### Current-clamp recordings

Whole-cell current-clamp recordings of TG neurons were made with the fast current-clamp mode of the Axopatch 200B amplifier by using a pipette solution of (in mM): 130 K gluconate, 10 NaCl, 2 MgCl_2_, 6 KCl, 14 Creatine phosphate, 4 MgATP, 0.3 GTP (Tris salt), and 10 HEPES, pH adjusted to 7.4 with KOH. The external solution contained (in mM): 145 NaCl, 5 KCl, 1 MgCl_2_, 2 CaCl_2_, 10 D-glucose, and 10 HEPES, with the pH adjusted to 7.4 with NaOH. Evoked action potentials were recorded using the current ramp protocol (300 pA in 0.5 s). The recorded membrane potentials were adjusted offline based on the calculated liquid junction potential of –15.5 mV.

### Voltage-clamp recordings

Whole-cell voltage-clamp recordings were performed on TG neurons using an extracellular solution containing (in mM): 120 Choline-Cl, 30 NaCl, 10 TEA-Cl, 10 D-glucose, 1 MgCl₂, 1 CaCl₂, 0.02 LaCl₃, and 10 HEPES, adjusted to pH 7.4 with TEA-OH. The internal pipette solution contained (in mM): 61 CsF, 61 CsCl, 9 NaCl, 1.8 MgCl₂, 9 EGTA, 10 HEPES, 14 Creatine phosphate, 4 MgATP, and 0.3 GTP (Tris salt), adjusted to pH 7.2 with CsOH. Cesium and lanthanum were included to block voltage-gated potassium and calcium channels, respectively. For recordings in HEK293T or ND7/23 cells expressing Na_V_ channels, the bath solution was (mM) 145 NaCl, 5 KCl, 1.8 CaCl₂, 1 MgCl₂, 10 D-Glucose and 10 HEPES (pH 7.4, NaOH), and the pipette solution (mM) 140 CsF, 10 NaCl, 1 EGTA, 14 Creatine phosphate, 4 MgATP, 0.3 GTP (Tris salt) and 10 HEPES (pH 7.2, CsOH). In experiments targeting hNa_V_1.8 in ND7/23 cells or TTX-R currents in TG neurons, 100 nM TTX was added to all external and working solutions to eliminate TTX-sensitive (TTX-S) sodium currents. In TG neurons, sodium currents were evoked by depolarizing steps from a holding potential of −80 mV to test potentials ranging from −80 mV to +10 mV in 10 mV increments. For hNa_V_1.7 and hNa_V_1.8 recordings, currents were evoked by depolarizing steps from a holding potential of −80 mV to test potentials ranging from −80 mV to +30 mV in 10 mV increments. Step durations were 100 ms for TTX-S and TTX-R current recordings.

To assess the voltage dependence of TTX-R sodium current activation in TG neurons, 100 ms depolarizing steps were applied from a holding potential of −80 mV to test potentials ranging from −80 mV to +30 mV in 10 mV increments. The voltage of half-maximal activation (V_0.5_) was estimated by calculating the macroscopic conductance (G) at each test potential using the extended Ohm’s law: *G* = *I*_peak_/(*V*_test –_ *V*_rev_), where *I*_peak_ is the peak current amplitude, *V*_test_ is the test potential, and *V*_rev_ is the apparent sodium reversal potential, determined individually for each cell. Conductance-voltage (G-V) relationships were then fitted with a Boltzmann function: *G* = *G*_max_/(1 + exp [(*V*_0.5_ – *V*_M_)/*k*]), where *G*_max_ is the maximal conductance, *V*_M_ is the membrane potential, V_0.5_ is the voltage at which half of the channels are activated, and k is the slope factor (in mV).

Availability curves of TTX-R sodium currents were obtained using a double-pulse protocol. A 120 ms conditioning prepulse was applied from a holding potential of −80 mV to voltages ranging from −120 mV to +20 mV in 10 mV increments, followed by a 20 ms test pulse to 0 mV. The peak current during the test pulse was normalized to the maximal response and plotted as mean ± SEM versus the prepulse voltage. The resulting data were fitted with a Boltzmann equation: *I*_test_/*I*_max_ = 1/(1+ exp[(*V – V*_0.5_)/*k*]), where *V* is the conditioning pulse voltage, *V*_*0.5*_ is the voltage at which half the channels are available, and *k* is the slope factor (in mV).

To evaluate the voltage dependence of activation for Na_V_ isoforms, 100 ms depolarizing steps were applied from a holding potential of −80 mV to test potentials ranging from −80 mV to +20 mV in 10 mV increments. For Na_V_1.8, the test range was extended up to +50 mV. The voltage of half-maximal activation (V_0.5_) was determined as explained above.

Availability curves for all Na_V_ isoforms were obtained using a double-pulse protocol. A 50 ms conditioning pulse from a holding potential of −80 mV was applied to voltages ranging from −120 mV to +20 mV in 10 mV increments, followed by a 30 ms test pulse to −10 mV. For Na_V_1.8, the test pulse was to +10 mV and extended to 100 ms. The peak current during the test pulse was normalized to the maximal current and plotted as mean ± SEM against the conditioning voltage. Data were fitted with a Boltzmann function as explained above.

A state-dependent block of hNa_V_1.8 current was established using the following protocol [20]: 10 s long conditioning pulses (*V*_cond_) were applied from a holding potential of −120 mV. The amplitude of the conditioning pulses varied systematically (in 10 mV steps) between -120 and 0 mV. Then, a 100 ms step to −120 mV was applied to remove fast inactivation, followed by a 5 ms test pulse (*V*_test_) to +10 mV. The resulting hNa_V_1.8 current amplitude was normalized to the maximal current amplitude (fraction available) and plotted (mean ± SEM) versus the voltage of conditioning pulses. The data were fitted using the Boltzmann equation, *I*_test_/*I*_max_ = 1/(1+ exp[(*V – V*_0.5_)/*k*]), where *V* is the conditioning pulse potential, *V*_0.5_ is the potential at which one-half of the channels are available. *k* is the slope factor (in mV).

A state-dependent block of hNa_V_1.7 current was assessed using the following protocol [8]: 5 s long conditioning pulses (*V*_cond_) were applied from a holding potential of −80 mV. The amplitude of the conditioning pulses varied systematically (in 10 mV steps) between –120 and −20 mV and was followed by a 10 ms test pulse (*V*_test_) to −10 mV. The resulting hNa_V_1.7 current amplitude was normalized to the maximal current amplitude (fraction available) and plotted (mean ± SEM) versus the voltage of conditioning pulses. The data were fitted using Boltzmann equation *I*_test_/*I*_max_ = 1/(1+ exp[(*V – V*_1/2_)/*k*]), where *V* is the conditioning pulse potential, *V*_1/2_ is the potential at which one-half of the channels are available, and *k* is the slope factor (in mV).

To evaluate the effects of THC on T-type calcium currents, the following solutions were used [21]: The extracellular solution contained (in mM): 10 BaCl₂, 152 tetraethylammonium chloride (TEA-Cl), and 10 HEPES, with the pH adjusted to 7.4 using TEAOH. The intracellular solution contained (in mM): 135 tetramethylammonium hydroxide, 10 EGTA, 40 HEPES, and 2 MgCl₂, with the pH adjusted to 7.2 using hydrofluoric acid.

T-type calcium currents were recorded by holding the membrane potential at −90 mV and applying depolarizing voltage steps from −80 mV to +60 mV in 10 mV increments, each lasting 250 ms. Current amplitude was determined by measuring the peak inward current and subtracting the current remaining at the end of the depolarizing pulse to minimize contamination from residual high-voltage–activated currents.

### Statistical analysis

The electrophysiological analysis was performed offline using Clampfit 10.7 software (Molecular Devices, USA). Fitting and statistical analysis were done using Prism 10 software (Graphpad Software Inc., USA). Data were compared by one or two-way analysis of variance (ANOVA) with the Bonferroni post hoc test and considered statistically significant when *p* ≤ 0.05. The box plots depict the mean and the 25th–75th percentiles, with whiskers indicating the minimum and maximum values. Otherwise, the data are presented as the mean ± SEM.

The count of action potentials in current-clamp recordings was established by detecting instances when the voltage crossed a specific threshold above the resting potential. Only neurons that demonstrated a stable resting potential and stable action potential threshold with no significant change in the action potential frequency-intensity relationship during the application of the extracellular solution were analyzed.

In voltage-clamped cells, concentration-response curves were calculated as the peak current amplitude evoked by depolarizing steps normalized to the control peak amplitude measured without drugs. Leak subtraction was applied before the normalization of the current amplitude. The peak currents were averaged and fitted to a non-linear sigmoidal concentration-response (variable slope) equation in GraphPad Prism software. Cells exhibiting drift or inconsistent baseline current were excluded from the analysis.

## Results

### THC suppresses action potential firing in nociceptor neurons

**Fig. 1:**
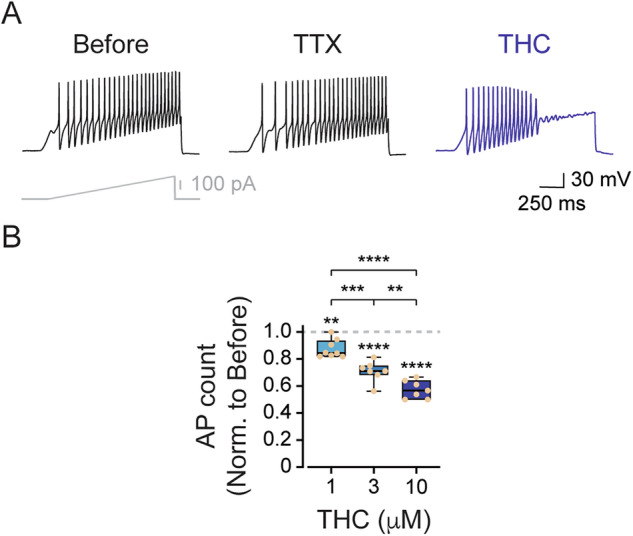
THC inhibits nociceptive firing. **A** Representative whole-cell current-clamp recording from acutely dissociated rat nociceptive TG neurons in response to a current ramp (300 pA in 1 s; *inset*) before (*left*), during exposure to 0.1 µM TTX (*middle*), and during exposure to 10 µM THC (*right*). **B** Concentration-response relationship for inhibition of the AP firing by THC in nociceptor TG neurons. Box plots and individual values demonstrate changes in the number of APs following 4 min of exposure to THC at the indicated concentrations. The number of action potentials was normalized to the number of evoked action potentials before the application of THC. One-way ANOVA, followed by Bonferroni’s post hoc test when **, *p* ≤ 0.01; ***, *p* ≤ 0.001; ****, *p* ≤ 0.0001.

To evaluate the direct effect of THC on nociceptor excitability, we performed whole-cell current-clamp recordings from acutely dissociated rat nociceptor (≤ 25 μm) trigeminal ganglion (TG) neurons. Neurons were stimulated with a ramp current protocol (300 pA over 1 s), reliably evoking repetitive action potentials under control conditions (Fig. 1A, left). As expected, the application of tetrodotoxin (TTX, 100 nM) slightly reduced firing, suggesting the expression of TTX-resistant sodium channels, a key feature of nociceptors [22–24] (Fig. 1A, middle). Notably, application of THC (10 μM) resulted in a robust suppression of action potential firing (Fig. 1A, right and Fig. S1A). This THC-induced inhibition of nociceptors’ firing was also significant at lower concentrations (i.e., 1 and 3 μM) (Fig. 1B). For experimental validation, we also analyzed the effect of CBD on nociceptors’ firing, and as previously reported [7, 25], CBD induced a pronounced inhibition of firing (Fig. S1B, C). These results demonstrate that THC directly reduces the excitability of nociceptive neurons.

### In nociceptor neurons, THC preferentially inhibits TTX-R sodium currents

**Fig. 2:**
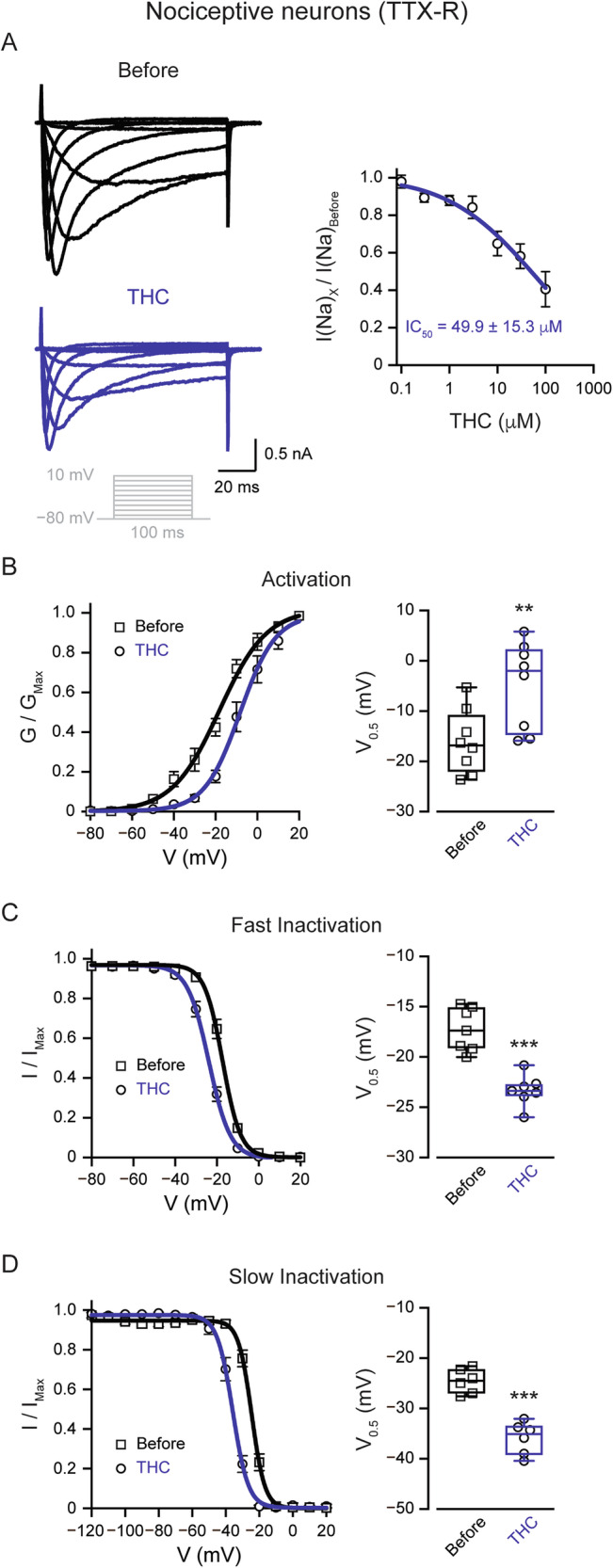
THC inhibits nociceptive sodium currents. **A** *Right:* Representative whole-cell voltage-clamp recording of TTX-R sodium currents from acutely dissociated rat nociceptive TG neurons before (*upper*) and after exposure to THC (10 µM; *lower*). Currents were elicited by depolarizing steps from a holding potential of −80 mV to 10 mV in 10 mV increments (*inset*). *Left:* Concentration-response relationship of TTX-R sodium currents amplitude (normalized to the current before the application of THC) following 4 min of exposure to THC at the indicated concentrations. The solid line represents the fit of the Hill equation. **B–C** *Left:* G/G_max_ (activation; (**B**) and I/I_max_ (availability; (**C**) curves for TTX-R sodium current before (*squares*) and 4 min after the application of 10 μM THC (*circles*). Note that THC induced a rightward shift in activation and a leftward shift in inactivation. To assess the voltage dependence of activation, 100-ms depolarizing steps were applied to a range of test potentials in 10 mV increments, from a holding potential of −80 mV to +30 mV. For the voltage dependence of fast inactivation, a double pulse protocol was used: a prepulse (V_cond_) was held constant at 120 ms and its amplitude was varied between −120 and +20 mV. I_test_ was assessed by stepping to 0 mV for 20 ms. The membrane was held at −80 mV. *Right:* Box plot and individual paired values of V_0.5_ of activation (**B**) and inactivation (**C**). Paired Student’s *t* test when **, *p* ≤ 0.01; ***, *p* ≤ 0.001. **D** *Left:* Voltage-dependence of TTX-R sodium currents steady-state channel availability (Fraction available, I/I_max_, plotted as a function of conditioning pulse voltage) before (*squares*) and 4 min (*circles*) after the application of 10 μM THC. V_cond_ was held constant at 10 s, and its amplitude was varied between −120 and 0 mV. 100 ms step to −120 mV was applied before V_test_. I_test_ was evoked by stepping to +10 mV for 5 ms. The membrane was held at −120 mV. Solid lines: fits to the Boltzmann function. Note a substantial decrease in channel availability following the treatment with THC. *Right:* Box plot and individual paired values of V_0.5_ of voltage-dependence of steady-state channel availability before and 4 min after the application of 10 μM THC. Paired Student’s *t* test when ***, *p* ≤ 0.001.

Because THC inhibits action potential firing despite the presence of TTX, it suggests that THC acts on TTX-resistant (TTX-R) sodium channels. TTX-R sodium currents provide the main inward current component of the action potentials and underlie the ability of nociceptors to fire repeatedly [22, 23, 26, 27]. Therefore, we first examine the effect of THC on the amplitude of TTX-R sodium current in nociceptor TG neurons. THC (10 μM) significantly reduced the peak TTX-R sodium current by ~35% (Fig. 2A). Application of THC led to a significant and prominent (~11 mV, *p* = 0.007, paired t-test, *n* = 8 neurons) rightward shift in the voltage dependence of activation of TTX-R currents (Fig. 2B). Moreover, it produced a significant leftward shift in the voltage dependence of fast inactivation of ~ 6 mV (*p* = 0.0004, paired t-test, *n* = 7 neurons, Fig. 2C). Notably, the application of THC substantially enhanced the slow inactivation of TTX-R current by significantly shifting its voltage dependence to the left by ~11 mV (*p* = 0.0002, paired t-test, *n* = 6 neurons) (Fig. 2D).

We next examined the effect of THC sodium current on large (≥35 μm) non-nociceptive TG neurons. These neurons express mainly TTX-S sodium channels [28, 29]. THC (10 μM) slightly but significantly reduced the peak of TTX-S sodium current by ~15% (Fig. S2A, B). Notably, the effect of THC on these currents was significantly smaller than on TTX-R currents in nociceptor neurons (Fig. S2C). Altogether, our results demonstrate that THC inhibits both TTX-R and TTX-S currents, but to a different extent. The higher efficacy of THC for TTX-R current implies a preferential effect of THC on nociceptor neurons.

### THC selectively inhibits Na_V_1.7 and Na_V_1.8 nociceptive sodium channels

**Fig. 3:**
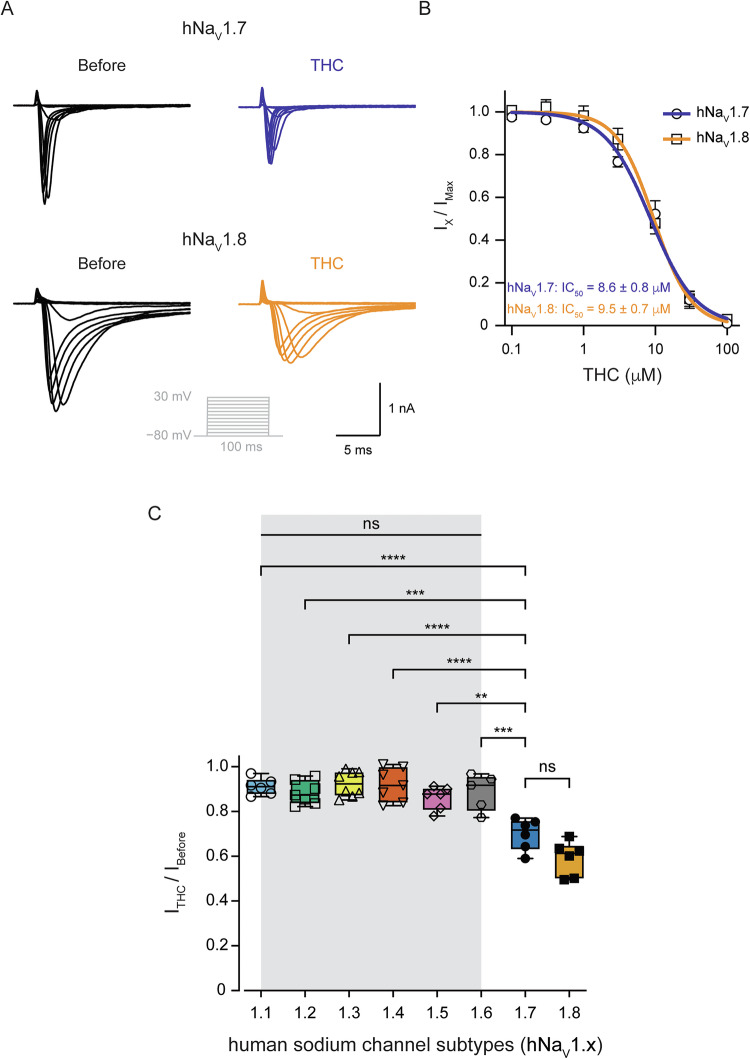
THC selectively inhibits the nociceptive hNa_V_1.8 and hNa_V_1.7 channels. **A** Representative whole-cell voltage-clamp recording from hNa_V_1.7 expressed in HEK293T cells (*upper*) and hNa_V_1.8 expressed in ND7/23 cells (*lower*) before (*left*) and after exposure to THC (10 µM; *right*). Currents were elicited by depolarizing steps from a holding potential of −80 mV to 30 mV in 10 mV increments (*inset*). **B** Concentration-response relationship for inhibition of hNa_V_1.7 (*circles*) and hNa_V_1.8 (*squares*) channels by THC. Each dot represents the mean and SEM of at least *n* = 6 cells. IC_50_ were determined by fitting Hill’s function to the data (shown as *blue and orange curves* for hNa_V_1.7 and hNa_V_1.8, respectively). **C** Box plots and individual values summarizing the inhibitory effect of 10 μM THC on the peak current of hNa_V_ channels. The current values are normalized to the value before the application of THC. Note that THC does not affect non-nociceptive specific sodium channel isoforms. One-way ANOVA, followed by Bonferroni’s post hoc test when ns not significant; **, *p* ≤ 0.01; ***, *p* ≤ 0.001; ****, *p* ≤ 0.0001.

Nociceptive neurons express both TTX-R Na_V_1.8 and TTX-S Na_V_1.7 isoforms [11]. Therefore, we examined the effect of THC on human Na_V_1.8 (hNa_V_1.8) and human Na_V_1.7 (hNa_V_1.7) expressed in heterologous systems. To express hNa_V_1.8, we used ND7/23 cells that enable the expression of this channel [30], and hNa_V_1.7 was expressed in HEK293T cells. The application of THC (10 μM) substantially reduced both hNa_V_1.8 and hNa_V_1.7 induced currents (Fig. 3A). The effect of THC on both channels was concentration-dependent, with similar potency and efficiency (Fig. 3B). These results demonstrate that THC is an inhibitor of Na_V_1.7 and Na_V_1.8 nociceptive sodium channels.

To further examine the specificity of THC to Na_V_1.7 and Na_V_1.8 channels, we analyzed its effect on all non-nociceptive specific sodium channels isoforms (hNa_V_1.1-1.6). In contrast to the non-psychoactive cannabinoid CBD [6], THC did not affect the amplitude of hNa_V_1.1-1.6 currents (Fig. 3C). Notably, THC demonstrates a significant inhibition of hNa_V_1.7-1.8 in comparison to other isoforms (Fig. 3C). No difference in THC effect between hNa_V_1.7 and hNa_V_1.8 was observed.

### THC inhibits Na_V_1.7 and Na_V_1.8 nociceptive sodium channels via the local anesthetic binding site

**Fig. 4:**
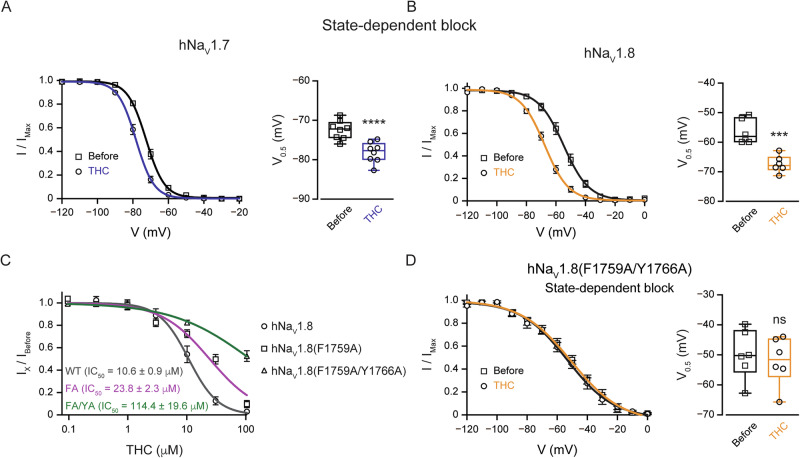
THC inhibits hNa_V_1.7 and hNa_V_1.8 channels via the local anesthetic binding site. **A** *Left:* Voltage-dependence of hNa_V_1.7 steady-state channel availability (I/I_max_), plotted as a function of conditioning pulse voltage before (squares) and after the application of THC (circles). Solid lines represent fits to a Boltzmann function. *Right:* Box plot and individual values of the half-inactivation potential (V_0.5_) before and after THC treatment. **B** Same as (**A**), but recorded from cells expressing hNa_V_1.8 channels before (squares) and after the application of THC (circles). Note the significant hyperpolarizing shift in the voltage dependence of inactivation for both channels, indicating that THC stabilizes the inactivated state. Paired Student’s *t* test when ***, *p* ≤ 0.001; ****, *p* ≤ 0.0001. **C** Concentration-response relationship for the inhibition of wild-type (WT) and mutated hNa_V_1.8 channels by THC. Data points represent the current inhibition for WT (circles; black line), the F1759A mutant (FA; squares; pink line), and the F1759A/Y1766A double mutant (FA/YA; triangles; green line) at various THC concentrations. The indicated IC_50_ values were determined by fitting Hill’s function to the data. Note the substantial rightward shift of the curves for the mutated channels, indicating a significant reduction in inhibitory potency. **D** Voltage-dependence of mutated hNa_V_1.8 (F1759A/Y1766A) steady-state channel availability (I/I_max_), plotted as a function of conditioning pulse voltage before (squares) and after the application of THC (circles). Solid lines represent fits to a Boltzmann function. *Right:* Box plot and individual values of the half-inactivation potential (V_0.5_) before and after THC treatment. Note that in the mutated hNa_V_1.8, THC does not lead to a change in the state-dependent block.

It has been shown that non-psychoactive cannabinoids inhibit sodium channels by stabilizing the inactivated state [6, 8, 10]. To examine whether THC acts via similar mechanisms, we tested whether THC induces a state-dependent block of Na_V_1.7 and Na_V_1.8 channels. We found that THC induces a significant left-ward shift of ~13 mV in the V_1/2_ of hNa_V_1.8 and a left-ward shift of ~6 mV in the V_1/2_ of hNa_V_1.7 (Fig. 4A, B). These results show that THC-induced inhibition of sodium channels is state-dependent, resembling local-anesthetic (LA) induced inhibition. Hence, we hypothesize that THC acts similarly to local anesthetics.

To examine this hypothesis, we tested whether classical mutations in this binding site of hNa_V_1.8 affect the THC inhibition [14]. We found that even a single F1759A mutation was sufficient to reduce the THC potency by about half a log (Fig. 4C). Adding the Y1766A mutation resulted in further reduction of THC potency to the level that the exact efficacy cannot be directly assessed (Fig. 4C). Moreover, the double LA binding site mutation abolished THC-induced state-dependent block (Fig. 4D). These data strongly suggest that THC acts through the LA binding site.

### hNa_V_1.8, but not hNa_V_1.7, has different susceptibility to phytocannabinoids

**Fig. 5:**
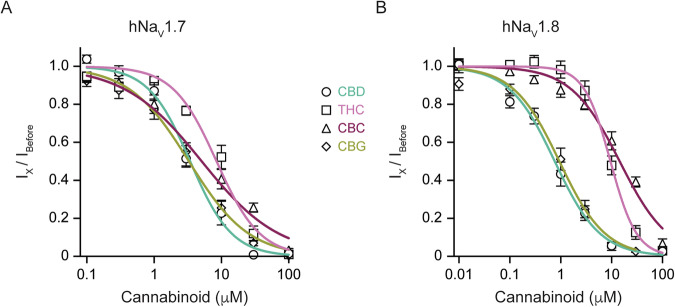
hNa_V_1.7 and hNa_V_1.8 channels exhibit differential sensitivity to phytocannabinoids. **A** Concentration-response curves for the inhibition of hNa_V_1.7 channels by cannabidiol (CBD; circles), Δ⁹-tetrahydrocannabinol (THC; squares), cannabichromene (CBC; triangles), and cannabigerol (CBG; diamonds). Peak current amplitude was normalized to the current before drug application (I_X_/I_Before_) and plotted against the cannabinoid concentration. Solid lines represent fits of the data to the Hill equation. Each data point represents the mean ± SEM from at least *n* = 6 cells. hNa_V_1.7: [CBD: IC_50_ = 3.4 ± 0.3 µM; THC: IC_50_ = 8.4 ± 0.8 µM; CBC: IC_50_ = 5.1 ± 0.7 µM; CBG: IC_50_ = 3.3 ± 0.3 µM]. **B** Same as (**A**), but for hNa_V_1.8 channels. hNa_V_1.8: [CBD: IC_50_ = 0.8 ± 0.1 µM; THC: IC_50_ = 9.5 ± 0.6 µM; CBC: IC_50_ = 14.4 ± 1.4 µM; CBG: IC_50_ = 0.9 ± 0.1 µM].

We next compared the effects of THC on hNa_V_1.7 and hNa_V_1.8 channels with those of the non-psychoactive phytocannabinoids cannabidiol (CBD), cannabigerol (CBG), and cannabichromene (CBC). We found that the effect of THC on hNa_V_1.7 was similar to that of non-psychoactive cannabinoids (Fig. 5A). However, the effect of THC on hNa_V_1.8 was similar to that of CBC but substantially weaker than the effect of CBD and CBG (Fig. 5B). Hence, our results suggest that while hNa_V_1.7 is affected similarly by all examined phytocannabinoids, hNa_V_1.8 discriminates between them.

## Discussion

The analgesic properties of cannabis are often attributed to the central actions of Δ⁹-tetrahydrocannabinol (THC), mediated by cannabinoid receptors [3, 31]. Here, we identify a distinct, non-canonical peripheral mechanism of action for THC, which may contribute to its analgesic efficacy. Our findings suggest that THC directly inhibits peripheral nociceptor excitability through the modulation of voltage-gated sodium channels hNa_V_1.7 and hNa_V_1.8. This suppression is facilitated by a direct, state-dependent interaction with the conserved local anesthetic (LA) binding site, establishing a peripheral analgesic pathway for THC that operates independently of cannabinoid receptor signaling.

The physiological significance of this mechanism is demonstrated by the suppression of action potential firing in nociceptive trigeminal ganglion neurons upon THC application. While our data suggest that THC’s efficacy in reducing action potential firing is lower than that reported for non-psychoactive cannabinoids such as CBD and CBG, its effect is nonetheless robust [6, 7, 9, 10] (Fig.S1). Moreover, we demonstrate that THC inhibits both TTX-S and TTX-R sodium currents. The smaller, yet significant, reduction of the TTX-S current in native nociceptors aligns with our finding that THC potently inhibits Na_V_1.7 while exerting no effect on other TTX-S isoforms (Na_V_1.1-1.6), thereby identifying Na_V_1.7 as the primary TTX-S target in these neurons.

Our investigation into specific sodium channel isoforms offers further clarification of this mechanism. The high selectivity that THC exhibits for the nociceptive channels hNa_V_1.7 and hNa_V_1.8 over other sodium channel isoforms is striking. However, previous studies have shown that Na_V_1.7 expression is broadly expressed across sensory neuron populations, including non-nociceptive neurons, with the highest levels in C-low-threshold mechanoreceptors [32]. In contrast, Na_V_1.8 exhibits more restricted expression, being predominantly found in nociceptors but also present in select mechanoreceptor populations [33]. Nevertheless, recent evidence from human DRG neurons [34] underscores the importance of the complementary interplay between Na_V_1.7 and Na_V_1.8 in defining nociceptor firing. Na_V_1.7 primarily governs the threshold and initial upstroke of the action potential, whereas Na_V_1.8 activates more gradually, considerably contributing to the peak amplitude and the characteristic shoulder of the action potential, and playing a critical role in maintaining repetitive firing [12, 26, 35, 36]. The dual inhibition of Na_V_1.7 and Na_V_1.8 by THC may provide greater therapeutic selectivity for nociceptors by leveraging their complementary roles in pain signal generation and transmission.

Our data demonstrate that THC affects neuronal and heterologously expressed sodium channels differently (compare Figs. 2A and 3B). This apparent mismatch could stem from the difference between the two experimental systems. The dose-response curve in Fig. 2A was generated from native neuronal preparations that contain a heterogeneous population of voltage-gated sodium channel subtypes. As we show in Fig. 3C, these subtypes have distinct sensitivities to THC. The resulting curve therefore represents the composite response of the entire population, plausibly obscuring the higher affinity of the most sensitive channel isoforms. In contrast, the experiment in Fig. 3B is performed on a homogeneous channel population (Na_V_1.7 or Na_V_1.8), revealing almost complete inhibition at 30 µM. Additionally, species-specific differences between the rodent channels in our native preparation and the human channels used for heterologous expression could contribute to the observed shift in potency.

Is the THC-induced inhibition of Na_V_1.7 and Na_V_1.8 the sole mechanism underlying its effect on nociceptor excitability? It has been shown that THC also inhibits T-type voltage-gated calcium channels (VGCC) [37, 38]. We also found that THC inhibits T-type VGCC in nociceptors, albeit with lesser potency than TTX-R channels (Fig. S3). These results imply that THC-induced inhibition of nociceptor excitability could result from its combined effect on Na_V_s and T-type calcium channels. Moreover, previous studies have demonstrated that primary nociceptor neurons express CB1R [39, 40]. Furthermore, we and others showed that THC directly activates TRPA1 channels that are also expressed in a subpopulation of nociceptors [41–44]. Consequently, THC may modulate neuronal excitability also via its effects on CB1R or TRPA1. However, whether these interactions will increase or decrease neuronal excitability (due to desensitization or depolarizing block) remains to be determined.

The leftward shift in the voltage dependence of both fast and slow inactivation induced by THC is characteristic of state-dependent channel blockers that preferentially stabilize inactivated states [45, 46]. This profile is consistent with the action of local anesthetics [47, 48]. Indeed, mutagenesis of key residues within the LA binding site of hNa_V_1.8 (F1759A and Y1766A) significantly attenuated the inhibitory effect of THC and abrogated its state-dependent properties. These data provide strong evidence that THC interacts directly with this conserved site, aligning the mechanism of a major phytocannabinoid with a classical pathway for local anesthesia.

Notably, the dose-response curve of THC’s effect on nociceptors’ TTX-R sodium currents is relatively shallow with a negative Hill slope of 0.50 ± 0.09 (Fig. 2A). Such a slope could result from THC’s effects on the heterogeneous population of neuronal channels. On the other hand, the apparent shallowness may not reflect the actual dose-response relation of THC, but a technical limitation of THC solubility rather than a pure biological effect. THC is highly hydrophobic (log P~7) with minimal aqueous solubility (~0.26 μg/mL) [49–52]. Hence, at high concentrations, THC precipitates out of solution via salt-out effects, preventing us from achieving a true pharmacological plateau. Consequently, our dose-response curve only partially captures the inhibition profile, resulting in an apparent Hill slope <1. This incomplete curve shape is well documented in studies of lipophilic drugs [53].

We found that the effects of THC on neuronal sodium currents occur at IC_50_ values in the high micromolar range (Fig. 2A). Are these doses physiologically relevant? Our results demonstrate a significant effect on neuronal firing already with 1 μM THC (Fig. 1). The IC_50_ values for the human sodium channels Na_V_1.7 and Na_V_1.8 are approximately 10 μM (Fig. 3). These nominal concentrations are indeed higher than the expected concentration of THC following cannabis consumption, which ranges between 0.1 and 1 μM within 3–10 min, coinciding with the onset of analgesia as reported in clinical trials [54–56]. However, the nominal aqueous concentrations used in our electrophysiological recordings do not directly reflect the effective concentrations at the lipid membrane-channel interface for a compound as lipophilic as THC. As previously demonstrated, THC partitions rapidly into cellular membranes, reaching local microenvironments in which its activity is substantially higher than that suggested by bulk solution levels [49, 57, 58]. Indeed, human pharmacokinetic and tissue-distribution studies demonstrate that after inhalation or vaporization, rapid redistribution of THC to lipid-rich tissues such as fat, skin, muscle, or nerve yields tissue/plasma ratios of 10–50, with measured local THC concentrations of 5–30 μM [56, 59–62]. These levels are well within the range required to produce Na_V_1.7/1.8 inhibition, as we showed in Fig. 3. Therefore, the direct modulation of peripheral nociceptor sodium channels can contribute to analgesia following THC consumption.

A comparison of THC with other phytocannabinoids under identical experimental conditions reveals significant pharmacological distinctions. Our findings demonstrate that, while hNa_V_1.7 is a relatively promiscuous target, being inhibited with comparable potency by THC, CBD, CBG, and CBC, hNa_V_1.8 functions as a molecular discriminator. This channel exhibited markedly lower sensitivity to THC and CBC in comparison to CBD and CBG. Such isoform-specific selectivity indicates that, despite sharing a common binding site, subtle structural variations among phytocannabinoids are sufficient to modify binding affinity within the hNa_V_1.8 channel. Specifically, THC and CBC both contain cyclic ether rings, whereas CBD features an open pyran ring, and CBG is an acyclic precursor [63]. It is well recognized that the rigid, closed pyran ring of THC is crucial for its high-affinity binding to the CB1 receptor and the subsequent psychoactive effects [31]. Consequently, it is plausible that these cyclic structures in THC and CBC may be less optimal for interaction with the LA binding site of Na_V_1.8 compared to the more flexible conformations of CBD and CBG. This observation enhances the understanding of the molecular pharmacology of the LA binding site and suggests potential directions for future structure-activity relationship studies aimed at designing cannabinoid derivatives with specific isoform selectivity.

In summary, this study provides evidence for an additional aspect of THC’s pharmacology. Besides its central receptor-mediated effects, THC also acts as a direct peripheral inhibitor of nociceptive sodium channels hNa_V_1.7 and hNa_V_1.8 through the local anesthetic binding site. Although it may be less potent than other phytocannabinoids in reducing overall firing, its high selectivity for Na_V_1.7 and Na_V_1.8 nociceptive sodium channels makes it an effective modulator of peripheral pain signaling. These findings help to clarify the peripheral analgesic effects of cannabis. Additionally, it suggests that developing peripherally restricted THC analogs could offer pain relief with fewer central nervous system side effects, opening a new potential direction for pain management strategies.

## Supplementary information

Supplementary information

## Data Availability

All data needed to evaluate the conclusions are presented in the paper. All the data and materials are fully available upon request from the corresponding authors.
