# Supplementary material for: The psychoactive cannabinoid THC inhibits peripheral nociceptors by targeting NaV1.7 and NaV1.8 nociceptive sodium channels
Source: Neuropsychopharmacology. 2026 Jan 21;51(6):1091–9. doi: 10.1038/s41386-026-02355-9 (PMC13125304; doi:10.1038/s41386-026-02355-9)
Supplement: Supplementary file 1 — Supplementary information [file 41386_2026_2355_MOESM1_ESM.pdf]

Supplementary information

The Psychoactive Cannabinoid THC Inhibits Peripheral Nociceptors by Targeting $Na_V1.7$ and $Na_V1.8$ Nociceptive Sodium Channels

Yossef Maatuf[1], Ariel Iskimov[1], Alexander M Binshtok[2,3*], and Avi Priel[1*]

[1]The Institute for Drug Research, School of Pharmacy, Faculty of Medicine, The Hebrew University of Jerusalem, Ein Karem, Jerusalem, 9112102, Israel.

[2]Department of Medical Neurobiology, Institute for Medical Research Israel-Canada, Faculty of Medicine, The Hebrew University of Jerusalem, Ein Karem, Jerusalem, 9112001, Israel.

[3]The Edmond and Lily Safra Center for Brain Sciences, The Hebrew University of Jerusalem, 911200, Israel.

*Co-corresponding author:

Avi Priel, PhD
avi.priel@mail.huji.ac.il

Alexander Binshtok, PhD
alexander.binshtok@mail.huji.ac.il

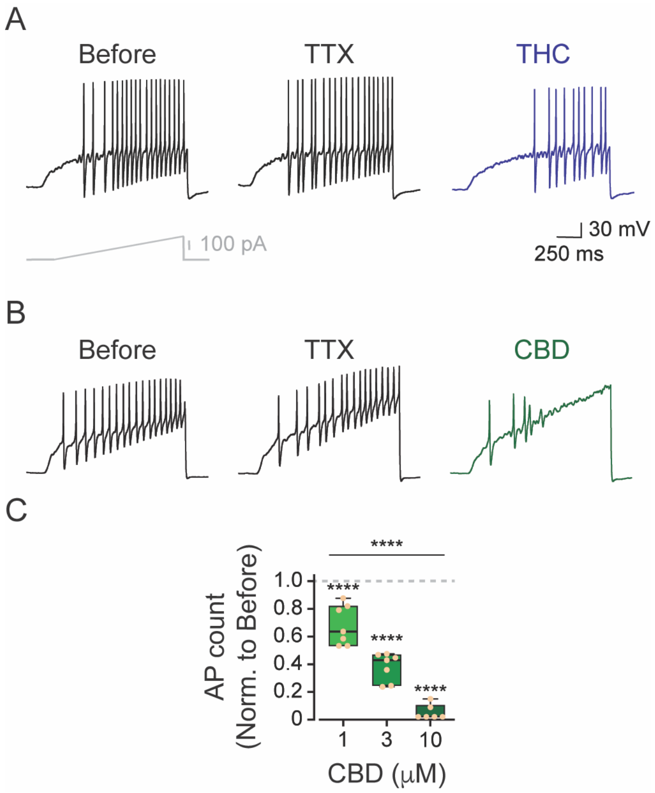

Figure S1. CBD inhibits nociceptive firing.

A and B. Representative whole-cell current-clamp recordings from acutely dissociated rat nociceptive TG neurons in response to a current ramp (300 pA in 1s; *inset*) before (*left*), during exposure to 0.1 μM TTX (*middle*), and during exposure to 10 μM THC (**A**; *right*) or 10 μM CBD (**B**; *right*).

C. Concentration-response relationship for inhibition of the AP firing by CBD in nociceptor TG neurons. Box plots and individual values demonstrate changes in the number of APs following 4 minutes of exposure to CBD at the indicated concentrations. The number of action potentials was normalized to the number of evoked action potentials before CBD application. One-way ANOVA, followed by Bonferroni's post hoc test when ****, $p \leq 0.0001$.

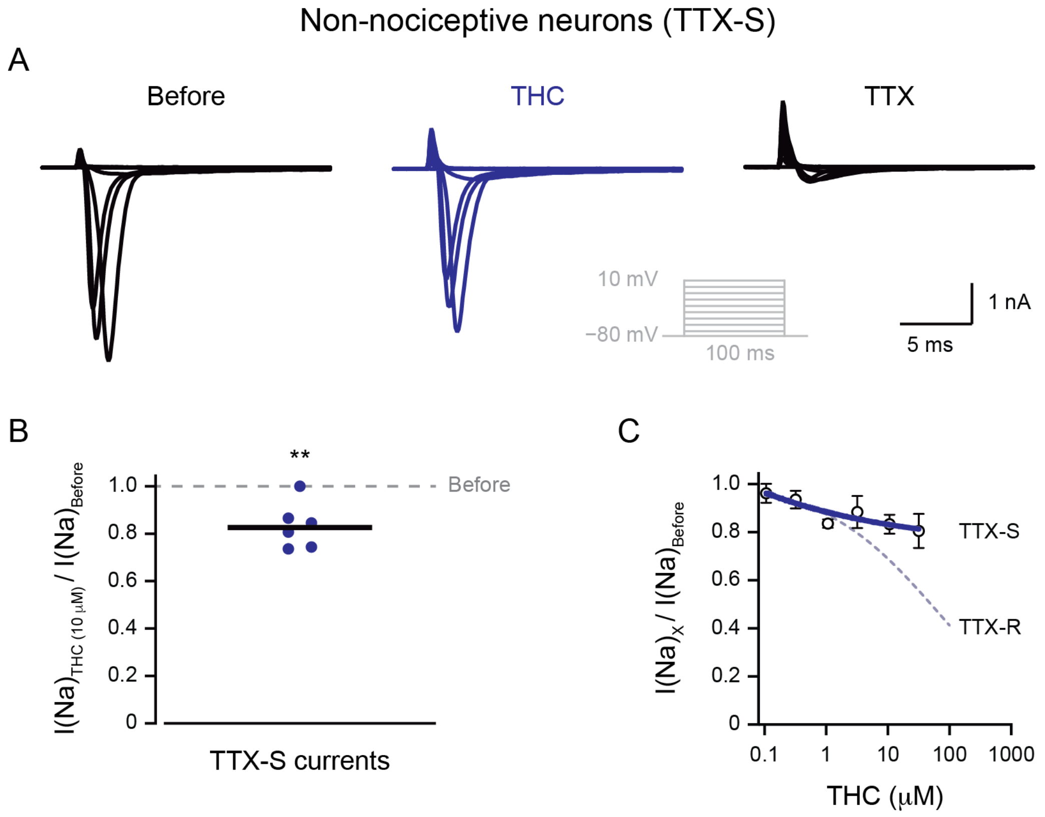

Figure S2. THC inhibits TTX-S sodium currents with lower efficacy than TTX-R

A. Representative whole-cell voltage-clamp recording of TTX-S sodium currents from acutely dissociated non-nociceptive rat TG neurons (≥ 35 μm) before (*left*) and after exposure to THC (10 μM; *middle*), and after TTX (0.1 μM; *right*) application. Currents were elicited by depolarizing steps from a holding potential of −80 mV to 10 mV in 10 mV increments (*inset*).

B. Mean and individual values of the sodium currents inhibition by 10 μM THC from non-nociceptive TG neurons (TTX-S) (*blue circles*). The grey dashed line represents the sodium current before the THC application. One-sample *t* test when **, $p \leq 0.01$.

C. Concentration-response relationship of TTX-S sodium currents amplitude (normalized to the current before the application of THC) following 4 minutes of exposure to THC at the indicated concentrations (solid blue line) and compared to the TTX-R currents (gray dashed line; from Fig. 2A).

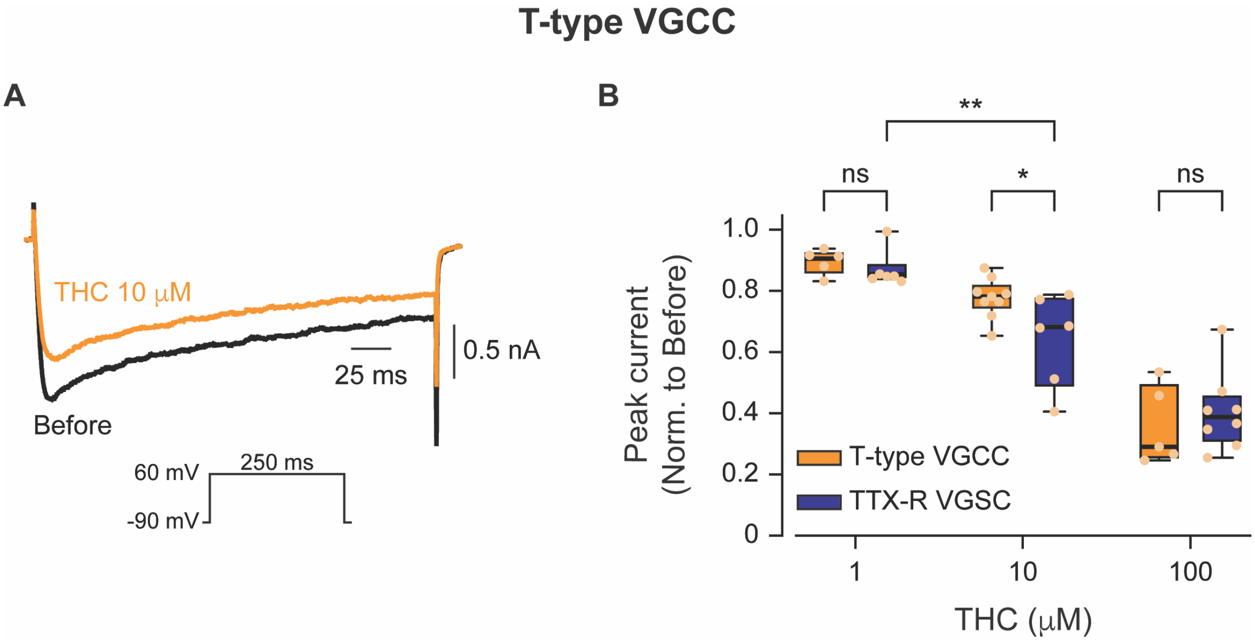

Figure S3. THC inhibits nociceptive T-type calcium currents less potently than TTX-resistant sodium currents, with a significant difference at 10 μM.

A. Representative traces of peak T-type calcium currents evoked by a 250 ms step to +60 mV from a holding of −90 mV before (black) and 10 min after the application of 10 μM THC (orange).

B. Box plot and individual values of the changes in the peak T-type current amplitude in TG nociceptor neurons with time (normalized to the current before the application of THC) following 10 minutes of exposure to THC at the indicated concentrations (orange) and compared to the THC effect on TTX-R currents in nociceptor TG neurons (blue). Two-way ANOVA was used to compare the effect of THC between the T-type and TTX-R currents (*, $p<0.05$; **, $p<0.01$; and ns – not significant). In all experiments, the number of dots represents the number of cells; one cell from the coverslip was recorded.
